# Alternative digital secondary education for marginalized, out-of-school youth in low- and middle-income countries: Protocol for a scoping review of intervention models, outcomes, and implementation challenges

**DOI:** 10.1371/journal.pone.0358760

**Published:** 2026-10-27

**Authors:** Jefferson Mwaisaka, Lilian Agesa, Ruth Nanjekho

**Affiliations:** Population Council, Nairobi, Kenya; Nanjing University, CHINA

## Abstract

**Background:**

Globally, 272 million children and youth were out of school in 2023, nearly half of whom are of secondary school age. Disparities disproportionately affect girls, displaced populations, and rural communities in low- and middle-income countries (LMICs). Digital technologies are increasingly promoted as alternative pathways to re-engage out-of-school youth in secondary education. However, evidence on intervention models, outcomes, and implementation challenges remains fragmented, with no comprehensive synthesis to inform policy and practice.

**Objective:**

This scoping review protocol aims to systematically map the available evidence on intervention models, outcomes, and implementation challenges of alternative digital secondary education for marginalized, out-of-school youth in LMICs.

**Materials and methods:**

This review will follow Joanna Briggs Institute methodology and PRISMA-ScR reporting guidelines. The protocol is registered on the Open Science Framework https://doi.org/10.17605/OSF.IO/5SRUD. A comprehensive search will be conducted across education, multidisciplinary, health, and regional databases, supplemented by grey literature from international organizations and preprint repositories. Eligible sources include empirical studies, program evaluations, policy documents, and reviews published from 2010 onwards – with no language restrictions, focusing on alternative digital secondary education for marginalized youth aged 13–24 years in LMICs. Two reviewers will independently screen studies and extract data using a standardized form, with a sample verified by a second reviewer. Findings will be synthesized using descriptive statistics and thematic analysis to identify patterns in intervention models, outcomes, implementation factors, and equity considerations.

**Conclusion:**

This review will provide the first comprehensive mapping of alternative digital secondary education for marginalized youth in LMICs, synthesizing fragmented evidence across sectors. Findings will inform evidence-based investments – guiding decisions on low-tech versus high-tech approaches, targeting of underserved populations, and priority areas for future evaluation. All data will be made publicly available on OSF. Protocol OSF Registration DOI https://doi.org/10.17605/OSF.IO/E8RZ3.

## Introduction

Quality education is a fundamental component of the United Nations’ Sustainable Development 2030 Agenda, which aims to ensure inclusive and equitable learning opportunities for all [1]. Yet for many children and youth in low- and middle-income countries (LMICs), quality education remains out of reach [2,3]. As of 2023, an estimated 272 million children and youth were out of school globally, with the largest burden in secondary education – 64 million in lower secondary and 130 million in upper secondary, accounting for nearly half (48%) of all out-of-school learners [4]. Regional disparities remain stark: 33% of school-aged children and youth in low-income countries are out of school, compared to just 3% in high-income countries, with over half of all out-of-school children and adolescents living in sub-Saharan Africa [3]. Access and inclusion barriers disproportionately affect girls, with gender disparities widening after primary school and negatively impacting enrollment and retention in secondary schools, and adult literacy outcomes [2,5–7]. These persistent inequities underscore the urgent need for alternative pathways to secondary education for marginalized, out-of-school youth.

For the purposes of this review, alternative digital secondary education refers to structured learning programs that provide secondary-level academic content to adolescents and youth who are out of formal schooling systems, and that are delivered primarily through digital or technology-enabled modalities. “Marginalized” refers to adolescents and youth who experience systemic exclusion from formal secondary education due to social, economic, geographic, or structural factors. This includes, but is not limited to, girls and young women, learners from low-income households, rural communities, ethnic or linguistic minorities, refugees and displaced populations, and youth with histories of interrupted or out-of-school learning. “Alternative” encompasses non-formal, community-based, NGO-led, government-supported open schooling, and hybrid models that operate outside conventional full-time, brick-and-mortar secondary schools. “Digital” includes both high-tech (online platforms, learning management systems, adaptive software) and low-tech (radio, television, SMS-based instruction) modalities where technology constitutes a central delivery mechanism. Secondary education refers to lower and/or upper secondary levels aligned with national curricula or credentialing pathways.

Digital transformation offers significant potential to expand access to quality and resilient learning systems [8,9], particularly for youth excluded from traditional schools due to social, geographical, or systemic barriers, or whose education has been disrupted by emergencies such as climate shocks or migration [8,10–12]. Evidence from diverse interventions highlights the promise of both high-tech platforms (online learning systems, adaptive software) and low-tech modalities (radio, television, SMS-based instruction) to reach underserved learners [12,13]. Realizing this potential, however, requires addressing persistent inequities in access to digital devices, content, and connectivity, as well as limitations in organizational readiness, educator preparedness, and community acceptance [11,14–17].

Alternative digital secondary education programs in LMICs face compounded challenges. Learners who faced challenges in face-to-face settings may experience even greater difficulty in digital contexts, particularly if they relied on services that become inaccessible in remote formats [18,19]. Educators are often unprepared for digital pedagogy, and communities may be skeptical of non-traditional learning pathways [15–17]. These factors affect not only recruitment but also retention, engagement, and the establishment of sustainable learning norms [18–20]. Limited or no face-to-face contact necessitates innovative strategies to support diverse learner needs, motivate students, and accommodate competing responsibilities in resource-constrained environments [19–21].

Pedagogical research indicates that effective digital learning depends on active engagement, constructive interaction, and foundational digital literacy for both learners and educators [22–24]. While these principles are well validated in high-resource contexts, their applicability to marginalized youth in LMICs, particularly those re-entering secondary education, remains uncertain. Structural disadvantage, interrupted schooling histories, and limited infrastructure may fundamentally alter how these pedagogies function in practice, highlighting the need for context-specific evidence. Importantly, for these learners, digital education is not an enhancement of existing schooling but an alternative pathway to re-engage with learning and obtain secondary education credentials. Given the diversity of program models, delivery modes, and outcome measures, a scoping review is particularly suited to systematically map the breadth and characteristics of existing evidence rather than estimate pooled effects. To date, no review has systematically mapped the intervention models, outcomes, and implementation challenges of alternative digital secondary education for marginalized, out-of-school youth in LMICs. This scoping review addresses this gap by examining how digital technologies are deployed and with what results, to advance inclusive and equitable quality secondary education for marginalized, out-of-school youth in LMICs, as envisioned by the 2030 Agenda.

## Objectives

The primary objective of this scoping review is to systematically map the available evidence on alternative digital secondary education interventions aimed at promoting school re-engagement and educational access among marginalized, out-of-school youth in low- and middle-income countries.

Specifically, this review seeks to answer the following research questions:

1. What digital and hybrid learning modalities (radio, television, mobile-based, online, offline, blended) are employed to deliver alternative secondary education to marginalized, out-of-school youth in LMICs?
2. What are the sociodemographic profiles (age, gender, socioeconomic status, geographic location) and educational histories (never enrolled, dropped out, displaced) of LMIC youth participating in these interventions?
3. What academic (learning gains, credential attainment), vocational (employment, skills training), and psychosocial (self-efficacy, well-being) outcomes are documented for participants?
4. What contextual, structural, and pedagogical factors facilitate or hinder the implementation, scalability, and sustainability of these interventions?
5. How do interventions address dimensions of digital equity, including access to devices and connectivity, digital literacy development, and accommodations for learners with disabilities or linguistic minorities, and what ethical considerations are raised?

## Review stages

This scoping review will be conducted in accordance with the Joanna Briggs Institute methodology for scoping reviews [25] and reported following the PRISMA Extension for Scoping Reviews (PRISMA-ScR) [S1 File] [26]. The protocol will be registered on the Open Science Framework (OSF) prior to commencing the full search.

### Stage 1: Preparation

The review questions were finalized as specified above. A data extraction form will be developed a priori, aligned with the five research questions. Eligibility criteria will be operationalized using the Population-Concept-Context (PCC) framework:

- Population: Marginalized, out-of-school youth aged 13–24 years in LMICs as defined by the World Bank classification [27], including refugees, displaced populations, and those never enrolled or who dropped out of secondary education. We selected the 13–24-year age range for two main reasons. First, it captures the overlap between commonly used definitions of adolescence and youth, including the United Nations definition of youth as persons aged 15–24 years, while also encompassing younger adolescents who are typically of secondary-school age. Second, the range reflects the realities of secondary education access in LMIC contexts, where over-age enrolment, grade repetition, delayed school entry, and interrupted schooling can result in learners accessing secondary-level education beyond the conventional secondary-school age range. The broad age range is therefore intended to reflect the diversity of learners who may require alternative pathways to secondary education rather than to imply that these age groups have equivalent developmental or educational needs. Differences across age groups will be accounted for through age-stratified reporting in the review synthesis, where sufficient information is available.
- Concept: Alternative digital secondary education interventions, defined as any structured learning program delivering secondary-level education partially or fully through digital means (radio, television, mobile, online, offline, or blended modalities) to out-of-school youth.
- Context: Low- and middle-income countries across all global regions, including humanitarian and crisis settings.

### Stage 2: Search

A comprehensive literature search will be conducted in the following electronic databases: Education Resources Information Center (ERIC ProQuest), Education Source (EBSCO), PubMed, Scopus, Web of Science, African Journals Online (AJOL), and the British Education Index. Gray literature sources will include organizational websites and repositories (UNESCO, UNICEF, World Bank, Inter-Agency Network for Education in Emergencies (INEE), Association for the Development of Education in Africa (ADEA); conference proceedings including Comparative and International Education Society (CIES) and the Education and Development Forum; and Preprint repositories including EdArXiv and SocArXiv.

Search strategies will combine terms related to: (1) population (“out-of-school,” “marginalized,” “disadvantaged”), (2) intervention (“digital,” “online,” “radio,” “mobile learning”), (3) education level (“secondary,” “high school”), and (4) geography (LMIC country names and regions). The search will cover the period from 2010 to present to capture literature following the expansion of mobile and low-tech educational interventions in LMICs. No language restrictions will be applied. Non-English sources will initially be translated using automated tools (DeepL or Google Translate) to support screening and data extraction. For all sources meeting the full-text eligibility criteria, the translated content will be reviewed by a team member or collaborator with native or professional proficiency in the source language, or, where appropriate, by a second analyst with sufficient proficiency in that language. This review will verify the accuracy of key extracted data and identify any translation errors, contextual nuances, or meanings that may be missed by automated translation. Search strategies will be refined iteratively and documented in full with all database-specific syntax included as appendices. All searched databases will be reported regardless of yield, with search dates and results documented using the data charting form (S2 File).

### Stage 3: Pilot screening

A pilot title and abstract screening will be conducted on a random subset of 50 records. Two reviewers will independently screen these records using Rayyan, with author names, journal titles, and publication year blinded where possible to minimize bias. If inter-rater agreement falls below 80%, eligibility criteria will be refined and the pilot repeated on a new subset until acceptable agreement is achieved.

### Stage 4: Protocol update

Any substantive changes to the eligibility criteria or screening approach identified during the pilot phase will be documented as an update to the OSF preregistration, with clear justification for modifications.

### Stage 5: Screening

All records from database and grey literature searches will be imported into EndNote 20 for deduplication, with automatic detection followed by manual verification. Deduplicated records will then be uploaded to Rayyan for screening. Screening will proceed in three sequential rounds: 1) Two independent reviewers will screen titles to remove clearly irrelevant studies; (2) remaining records will be screened independently by two reviewers based on abstracts and keywords; (3) full texts of potentially eligible studies will be retrieved and independently assessed by two reviewers against the inclusion criteria. At each round, records deemed potentially relevant by either reviewer will proceed to the next stage. Discrepancies at any stage will be resolved through discussion or, if necessary, consultation with a third reviewer. Reasons for exclusion at full-text stage will be documented and reported in the final PRISMA-ScR flow diagram.

### Stage 6: Pilot data extraction

A pilot data extraction will be conducted on approximately 10 included sources to test the data extraction form. The form will capture:

- Bibliographic information (author, year, country, source type)
- Intervention characteristics (modality, duration, curriculum, provider)
- Population characteristics (sample size, age, gender, educational history)
- Outcomes reported (academic, vocational, psychosocial)
- Implementation factors (barriers, enablers, scalability)
- Equity considerations (digital access provisions, accommodations for learners with disabilities, and linguistic support)
- Ethical issues discussed

Reviewers will extract the same 10 sources independently and compare results. The form will be refined based on discrepancies or missing fields before full extraction begins.

### Stage 7: Protocol update

Any modifications to the extraction framework based on pilot findings will be transparently documented in a subsequent OSF update.

### Stage 8: Data extraction

Data will be extracted from all included studies using the finalized extraction form. One reviewer will perform full data extraction, while a second reviewer will independently verify accuracy for a randomly selected 20% sample of included studies. This 20% proportion balances feasibility with reliability, consistent with scoping review best practices [28]. Any discrepancies identified during verification will be resolved through discussion between the two reviewers.

### Stage 9: Synthesis

Extracted data will be synthesized in three phases:

- Descriptive numerical summary – Frequencies and percentages will be calculated for study characteristics (geographic distribution, publication year, intervention modalities, study designs) and will be presented in tables and figures.
- Thematic analysis – Extracted data on outcomes, implementation determinants, and equity considerations will be analyzed using Braun & Clarke (2023) [29] reflexive thematic analysis approach. Hybrid (inductive and deductive) coding will be used. Two reviewers will independently code five included studies using NVivo, develop preliminary themes, and iteratively refine them through discussion until consensus is reached. If disagreement persists, a third reviewer will be consulted. Themes will be organized around the review’s core foci: intervention models, outcomes, and implementation challenges.
- Narrative synthesis: Findings will be presented narratively, integrating descriptive statistics and thematic patterns to map the current evidence landscape.

### Stage 10: Reporting and dissemination

Results will be reported in accordance with PRISMA-ScR guidelines and disseminated through peer-reviewed publication, conference presentations, and stakeholder summaries targeting education policymakers and practitioners in LMICs.

## Eligibility criteria

The eligibility criteria will be guided by the Population – Concept - Context (PCC) framework.

### Inclusion criteria

- Participants: Marginalized youth aged 13–24 years in LMICs (as defined by the World Bank) who are out of formal secondary education, including those who never enrolled, dropped out, or are displaced (refugees, crisis-affected populations). “Marginalized” encompasses socioeconomic disadvantage, ethnic or linguistic minority status, disability, geographic remoteness, gender-based exclusion, or other forms of systemic educational exclusion.
- Concept: Alternative digital secondary education interventions, defined as structured learning programs that deliver secondary-level academic content partially or fully through digital or technology-enabled modalities (online platforms, mobile learning, radio, television, SMS-based instruction, blended models) to out-of-school youth for the purpose of educational re-engagement and credentialing.
- Context: Studies conducted in LMICs across all global regions as defined by the World Bank (2026), including humanitarian and crisis settings.
- Types of Evidence Sources: Empirical studies (quantitative, qualitative, mixed-methods), systematic and scoping reviews, program evaluations, policy documents, and grey literature (NGO reports, government publications, academic dissertations) that report on intervention characteristics, outcomes, or implementation factors. Systematic and scoping reviews will be included to map the existing review landscape and identify additional primary studies but will not be double-counted in the synthesis of intervention findings.
- Language: No language restrictions will be applied. Non-English sources will initially be translated using automated tools (DeepL or Google Translate) to support screening and data extraction. For sources meeting the full-text eligibility criteria, translated content will be reviewed by a team member or collaborator with native or professional proficiency in the source language, or by a second analyst with sufficient proficiency in that language, to verify the accuracy of key extracted data and identify potential translation errors or contextual nuances.
- Publication Date: Studies published from 2010 onwards to capture literature following the expansion of mobile and low-tech educational interventions in LMICs.

### Exclusion criteria

- Studies focused primarily on general workforce development, technical/vocational training without an educational re-engagement or secondary education component.
- Opinion pieces, editorials, and conference abstracts without accompanying full papers
- Studies that do not report on intervention characteristics, outcomes (academic, vocational, psychosocial), or implementation factors relevant to the research questions.

## Search strategy

This scoping review will employ a comprehensive, multi-method search strategy developed in accordance with the Joanna Briggs Institute methodology for scoping reviews [25] and the PRISMA-ScR reporting guidelines [26]. The search will be executed from April to May 2026 and documented in full to ensure transparency and reproducibility.

The following electronic databases will be systematically searched: Education Resources Information Center (ERIC), Education Source, PsycINFO, Scopus, Web of Science Core Collection, PubMed/MEDLINE, Google Scholar, African Journals Online (AJOL), and the British Education Index. Grey literature sources will be systematically searched following established guidance [30], and will include ProQuest Dissertations and Theses Global and the Networked Digital Library of Theses and Dissertations (NDLTD) for unpublished theses and dissertations; organizational repositories including the UNESCO Digital Library, UNICEF Innocenti Research Centre Publications, World Bank Open Knowledge Repository, Inter-Agency Network for Education in Emergencies (INEE) Resource Database, and Association for the Development of Education in Africa (ADEA) Publications; conference proceedings from the Comparative and International Education Society (CIES) and the UKFIET International Conference on Education and Development; and preprint repositories including EdArXiv and SocArXiv. To ensure comprehensive coverage, backward citation searching will be conducted by screening the reference lists of all studies included after full-text screening, as well as reference lists of relevant systematic and scoping reviews [31]. Forward citation searching will be conducted for key included studies using Scopus and Web of Science citation tracking functionality to identify subsequent research that has cited them.

### Search term development

Search strategies will be developed iteratively and will combine controlled vocabulary (database-specific subject headings) with free-text keywords across three conceptual domains derived from the Population – Concept – Context framework. Boolean operators (OR, AND) will be applied, and truncation (*) will be used to capture variant spellings and plural forms. Where database functionality permits, field limitations will be applied to title, abstract, and subject heading fields.

For the population domain, controlled vocabulary will include ERIC descriptors “Out of School Youth,” “Dropouts,” “Disadvantaged Youth,” and “At Risk Students”; PsycINFO subject headings “At Risk Populations” and “School Dropout”; and PubMed MeSH terms “Adolescent,” “Young Adult,” and “Refugees.” Free-text keywords will include “out-of-school youth,” “school dropout,” “never enrolled,” “displaced youth,” “refugee youth,” “marginalized youth,” “at-risk youth,” “adolescen,” and “young adult.”

For the concept domain, controlled vocabulary will include ERIC descriptors “Educational Technology,” “Electronic Learning,” “Blended Learning,” “Distance Education,” “Nonformal Education,” and “Open Education”; PsycINFO subject headings “Computer Assisted Instruction” and “Distance Education”; and PubMed MeSH terms “Educational Technology,” “Computer-Assisted Instruction,” and “Mobile Applications.” Free-text keywords will include “digital education,” “e-learning,” “online learning,” “blended learning,” “mobile learning,” “radio instruction,” “educational radio,” “television instruction,” “SMS-based,” “low-tech,” “alternative education,” “second chance education,” “open schooling,” and “non-formal education.”

For the context domain, controlled vocabulary will include ERIC descriptors “Developing Nations” and “Low Income Countries”; PubMed MeSH term “Developing Countries”; and PsycINFO subject heading “Developing Countries.” Free-text keywords will include “low-income countr,” “middle-income countr,” “LMIC,” “developing countr,” “Global South,” “sub-Saharan Africa,” “Asia,” “Latin America,” and the names of all countries currently classified as low- or middle-income by the World Bank, verified at the time of search execution.

### Search documentation and reporting

All searches will be documented in accordance with PRISMA-ScR guidelines [26]. For each database and grey literature source, the following information will be recorded: database or source name and platform, date of search execution, complete search strategy including all syntax and limiters, and number of results retrieved. Full search strategies for all databases will be documented and reported as part of the study methods. Search results will be reported in a PRISMA-ScR flow diagram, including records identified through database searching, grey literature sources, and supplementary methods; duplicates removed; records screened at title and abstract level; full-text articles assessed for eligibility; full-text articles excluded with reasons; and studies included in the final synthesis.

### Study selection process

All identified citations will be exported from each database in standard format and imported into Rayyan for deduplication and screening [32]. Two independent reviewers will screen titles and abstracts against the eligibility criteria. All records deemed potentially relevant by either reviewer will proceed to full-text review. Full texts will then be independently assessed by two reviewers. Discrepancies at either stage will be resolved through discussion or, if necessary, consultation with a third reviewer. Reasons for exclusion at full-text stage will be documented and reported in the final PRISMA-ScR flow diagram.

### Data charting

A standardized data charting form (S2 File) will be developed in Microsoft Excel and pilot-tested on approximately 10 included sources, consistent with Stage 6. The form will be refined iteratively based on pilot findings to ensure alignment with the review objectives and consistency between reviewers. From each included source, the following data will be charted:

- Bibliographic information: Author(s), year of publication, title, source type (journal, report, dissertation, conference paper), country or region.
- Study characteristics: Study design, sample size, participant characteristics (age, gender, educational history), setting (urban/rural, school/community/household), and duration of intervention or data collection.
- Intervention characteristics: Type of digital education intervention, delivery modality (online, mobile, radio, television, SMS, blended), technology or platform used, and implementation context.
- Outcomes reported: Academic (re-enrollment, learning gains, completion), psychosocial (self-efficacy, motivation, well-being), vocational (employment, skills training), or other outcomes relevant to school re-engagement.
- Implementation and contextual factors: Reported enablers, barriers, equity considerations (digital access, accommodations for disability or linguistic minorities), and contextual influences on implementation.
- Other key findings: Additional quantitative results or qualitative themes relevant to the review questions not captured under outcomes.
- Methodological characteristics: Study limitations reported by authors and funding sources.

Data charting will be conducted by one reviewer and verified by a second reviewer for accuracy and completeness on a randomly selected 20% sample of included studies, consistent with Stage 8. Any discrepancies will be resolved through discussion. In line with JBI guidance for scoping reviews, a formal risk of bias assessment will not be conducted; however, methodological characteristics will be descriptively reported to contextualize findings. When key information is not reported in the original source, the variable will be coded as “not reported.” These reporting gaps will be documented and described as part of the evidence mapping process.

## Data management and sharing

All records identified through database and grey literature searches will be imported into EndNote 20 for reference management and deduplication. Screening decisions at title, abstract, and full-text stages will be managed using Rayyan, with records exported as CSV files for documentation and reproducibility. A standardized data charting spreadsheet (S2 File) will be maintained in Microsoft Excel to record extracted study characteristics and findings. All datasets generated during the review, including bibliographic records, screening decisions, and the final data charting dataset, will be made publicly available through the Open Science Framework upon publication of the review. Data files will be shared in machine-readable formats (CSV/XLSX) with accompanying documentation to support transparency and reuse. Personal communications with study authors and other confidential materials will not be publicly shared.

### Data quality

Consistent with guidance from the JBI Manual for Evidence Synthesis and reporting standards outlined in PRISMA-ScR [26], a formal risk-of-bias assessment will not be conducted, as the purpose of this review is to map the range and characteristics of available evidence rather than evaluate comparative effectiveness. Prior to data extraction, the publication status of all included sources will be verified using the Retraction Watch database to identify any retractions, expressions of concern, or major corrections; retracted studies will be excluded. For grey literature reports, corroborating publications or official project documentation will be sought to verify basic descriptive facts, with discrepancies documented and the most authoritative source prioritized. To ensure accuracy and consistency of extracted data, a randomly selected 20% sample of included sources will undergo dual independent extraction by a second reviewer, as described in Stage 8. Discrepancies will be resolved through discussion and, if necessary, consultation with a third reviewer.

To contextualize the evidence base, each included source will be described according to study design, reporting completeness (presence of key methodological details), and source type (peer-reviewed, grey literature). Sources will not be excluded based on these characteristics; instead, the methodological landscape will be transparently described to enable readers to interpret findings with appropriate caution. All validation steps and decisions will be recorded in the extraction template to ensure transparency and reproducibility.

### Data synthesis plan

This scoping review employs a descriptive-analytical synthesis to map the evidence. The procedure is tiered, moving from descriptive tabulation to thematic analysis, aligning directly with the research questions.

Given the broad age range of the target population (13–24 years), participant age and age-related characteristics will be extracted from all included studies where reported. To account for developmental and educational differences across this range, findings will be descriptively reported by age band where sufficient disaggregated information is available. The planned age bands are: early adolescence (13–15 years), mid-to-late adolescence (16–19 years), and young adulthood (20–24 years). Age-stratified reporting will be applied to key review domains, including intervention characteristics, educational outcomes (academic, social-emotional, vocational/employment) and other reported outcomes, and implementation factors. Where studies include participants spanning multiple age bands but do not provide disaggregated findings, this will be documented and findings will be reported for the study population as a whole. We will not exclude studies solely because they include mixed-age populations, provided they otherwise meet the eligibility criteria. This approach will allow the review to capture the breadth of alternative digital secondary education models while recognizing potential differences in intervention needs, outcomes, and implementation challenges across developmental and age groups.

Additionally, given the substantial evolution of educational technologies over the review period, from low-tech and basic mobile tools to more advanced digital, adaptive, and AI-enabled models, the technological characteristics of each intervention will be systematically extracted and classified. The data-charting form will include a dedicated Platform/Technology field to capture the primary technology or technologies used to deliver the intervention. For descriptive synthesis, interventions will be broadly categorized as: (1) low-tech/offline, including SMS, radio, and television-based approaches; (2) basic digital/online, including static e-learning platforms, learning management systems, and recorded digital content; and (3) advanced/adaptive/AI-enabled, including adaptive learning software, AI-enabled tutoring systems, and algorithm-personalized content. Where an intervention incorporates multiple technologies, the key technologies and their respective roles in intervention delivery will be recorded. The publication year of each included study will also be extracted and reported alongside its technological category. This will allow the review to descriptively examine the evolution of alternative digital secondary education models over the review period and to contextualize differences in intervention characteristics, educational outcomes, and implementation challenges according to the technological modalities employed. Relevant infrastructure and access requirements, including internet connectivity, electricity, and device requirements, will also be extracted where reported.

#### Tier 1: Descriptive numerical synthesis.

We will use Microsoft Excel to generate frequency counts and percentages for categorical data extracted across all included sources. This will produce summary tables and charts depicting:

- The distribution of intervention types and technological platforms (RQ1).
- The frequencies of targeted marginalized populations and their demographic characteristics (RQ2).
- The prevalence of reported outcome domains (academic, social-emotional, vocational/employment) (RQ3).
- The geographic spread of the evidence.

#### Tier 2: Thematic narrative synthesis.

Extracted qualitative and descriptive data on implementation factors, barriers, equity considerations and ethics will be imported into NVivo. We will conduct a qualitative content analysis using a combined deductive-inductive approach.

- Deductive Phase: Data will initially be coded into broad categories pre-defined by the research questions (“Barrier - Infrastructure,” “Enabler - Community Involvement,” “Ethical Concern - Data Privacy”).
- Inductive Phase: Within these categories, we will perform open coding to identify specific, emergent themes and sub-themes (under “Infrastructure,” codes may emerge for “intermittent electricity,” “high data cost,” “device sharing challenges”).
- Synthesis: Themes will be analyzed for relationships, patterns, and dissonance across different contexts or intervention types. This will be used to build a coherent narrative summary.

#### Tier 3: Evidence gap and conceptual mapping.

We will synthesize findings from Tiers 1 and 2 to create a conceptual map – a matrix or diagram – that visually links intervention types, target populations and outcomes measured. This will be supplemented by a narrative summary of clear gaps, such as populations or outcome domains unaddressed by current interventions, or missing study designs such as lack of cost-effectiveness analyses. If the volume or heterogeneity of qualitative data is too great for in-depth thematic analysis with the available resources, the synthesis will default to a structured narrative summary organized by the deductive categories, explicitly noting the limitation. Additionally, if data for a specific research question, for example, RQ5 on ethical considerations is extremely sparse, we will report this as a primary finding, an evidence gap, rather than attempting a forced synthesis.

## Discussion

This scoping review will provide the first systematic mapping of alternative digital secondary education interventions for marginalized, out-of-school youth in LMICs, following JBI methodology and PRISMA-ScR guidance. Through synthesis of evidence across education, development, technology, and humanitarian sectors, the review will clarify the range of intervention models, target populations, outcomes, and implementation factors. Findings are expected to guide decisions on low- versus high-tech approaches, strategies to reach underserved populations, and priority areas for evaluation or scale-up. Potential limitations include heterogeneity of study designs, variable reporting quality, and incomplete data in grey literature. Despite these challenges, the review will provide a transparent overview of the current evidence landscape and identify critical gaps to inform future research, program development, and policy in alternative digital secondary education for marginalized youth.

## Supporting information

S1 File PRISMA-ScR checklist. Completed PRISMA Extension for Scoping Reviews (PRISMA-ScR) checklist, with page numbers indicating where each item is addressed.
(DOCX)

S2 File Data charting form. Blank data charting form (Microsoft Excel) used for pilot testing and full extraction, including all categories: bibliographic information, study characteristics, intervention characteristics, outcomes reported, implementation and contextual factors, qualitative findings, and methodological characteristics.
(XLSX)
