# Supplementary material for: Alternative digital secondary education for marginalized, out-of-school youth in low- and middle-income countries: Protocol for a scoping review of intervention models, outcomes, and implementation challenges
Source: PLoS One. 2026 Oct 27;21(10):e0358760. doi: 10.1371/journal.pone.0358760 (PMC13712531; doi:10.1371/journal.pone.0358760)
Supplement: Supplementary file 2 [file pone.0358760.s004.docx]

**Response to Reviewers**

**Alternative digital secondary education for marginalized, out-of-school youth in low- and middle-income countries: protocol for a scoping review of intervention models, outcomes, and implementation challenges**

We sincerely thank the reviewers and editor for their thoughtful and constructive feedback on our manuscript. We have carefully considered all comments and revised the manuscript accordingly to improve its clarity and scientific rigor. In the sections that follow, we provide detailed, point-by-point responses to each comment

**Journal Requirements:**

1. Please ensure that your manuscript meets PLOS ONE’s style requirements, including those for file naming.

- **Response:** Thank you for this reminder. We have reviewed the manuscript file names, formatting, and reference style against PLOS ONE’s current author guidelines and made the necessary adjustments in the manuscript to ensure compliance.

2. When completing the data availability statement of the submission form, you indicated that you will make your data available on acceptance. We strongly recommend all authors decide on a data sharing plan before acceptance, as the process can be lengthy and hold up publication timelines. Please note that, though access restrictions are acceptable now, your entire data will need to be made freely accessible if your manuscript is accepted for publication. This policy applies to all data except where public deposition would breach compliance with the protocol approved by your research ethics board. If you are unable to adhere to our open data policy, please kindly revise your statement to explain your reasoning and we will seek the editor’s input on an exemption. Please be assured that, once you have provided your new statement, the assessment of your exemption will not hold up the peer review process.

- **Response:** Thank you for raising this. As this is a protocol for a scoping review, the study does not involve primary data collection from human participants or any data subject to ethical or licensing restrictions. The protocol has been registered on OSF - <https://doi.org/10.17605/OSF.IO/E8RZ3>, and all data extracted during the review (from published, publicly accessible sources) will be made freely available at the time of publication of the review results via the same OSF project. We have revised our Data Availability Statement accordingly.

3. Please include captions for your Supporting Information files at the end of your manuscript, and update any in-text citations to match accordingly. Please see our Supporting Information guidelines for more information: <http://journals.plos.org/plosone/s/supporting-information>.

- **Response:** Thank you for this guidance. We have added captions for both Supporting information files at the end of the manuscript, after the References section, as per PLOS ONE formatting requirements. We have also checked that in-text citations to S1 File (Line 157) and S2 File (Lines 410 & 447) match accordingly.

4. If the reviewer comments include a recommendation to cite specific previously published works, please review and evaluate these publications to determine whether they are relevant and should be cited. There is no requirement to cite these works unless the editor has indicated otherwise.

- **Response:** Thank you for this clarification. No specific previously published works were recommended for citation in the reviewer comments; therefore, no additional references were added.

5. Please review your reference list to ensure that it is complete and correct. If you have cited papers that have been retracted, please include the rationale for doing so in the manuscript text, or remove these references and replace them with relevant current references. Any changes to the reference list should be mentioned in the rebuttal letter that accompanies your revised manuscript. If you need to cite a retracted article, indicate the article’s retracted status in the References list and also include a citation and full reference for the retraction notice.

- **Response:** Thank you for this guidance. We have reviewed the reference list and confirm that none of the cited works have been retracted. No changes to the reference list were therefore required as a result of this review.

**Review Comments to the Author**

Reviewer #1: General Comments: The paper is very well written and targets crucial gap in the current literature about alternative digital secondary education for marginalized, out-of-school youth in LMICs. Using the JBI methodology and the PCC framework is a good method and gives a strong foundation for this scoping review but I have a few specific concerns that the authors should address to upgrade the paper.

1. Range of age: The chosen age of 13 to 24 years feels too wide. The academic and cognitive demands of a 13-year-old kid are totally different from those of a 24-year-old mature person. The writers require to interpret why they selected such a wide range, or they likely to plan a sub category analysis to divide these different age groups.
   - **Response:** Thank you for this important observation. We selected the 13-24-year age range for two main reasons. First, it captures the overlap between commonly used definitions of adolescence and youth, including the United Nations definition of youth as persons aged 15-24 years, while also encompassing younger adolescents who are typically of secondary-school age. Second, the range reflects the realities of secondary education access in LMIC contexts, where over-age enrolment, grade repetition, delayed school entry, and interrupted schooling can result in learners accessing secondary-level education beyond the conventional secondary-school age range. The broad age range is therefore intended to reflect the diversity of learners who may require alternative pathways to secondary education, rather than to imply that these age groups have equivalent developmental or educational needs. We have added this clarification to the manuscript (Lines 170-181).
   - To account for developmental and educational differences across this age range, participant age and age-related characteristics will be extracted from all included studies where reported. Findings will be descriptively reported by age band where sufficient disaggregated information is available. The planned age bands are early adolescence (13-15 years), mid-to-late adolescence (16-19 years), and young adulthood (20-24 years). Age-stratified reporting will be applied across key review domains, including intervention characteristics, educational outcomes (academic, social-emotional, and vocational/employment), other reported outcomes, and implementation factors. Where studies include participants spanning multiple age bands but do not provide disaggregated findings, this will be documented and findings will be reported for the study population as a whole. Studies will not be excluded solely because they include mixed-age populations, provided they otherwise meet the eligibility criteria. This approach will allow the review to capture the breadth of alternative digital secondary education models while recognizing potential differences in intervention needs, outcomes, and implementation challenges across developmental and age groups. We have added this clarification to the Data synthesis plan section of the manuscript (Lines 481-495).
2. Risk of translation: The paper mentions depending on automated tools like DeepL or Google Translate for reviews not written in English. This creates risk of translation errors or missing the actual meaning. To ensure accuracy, Please confirm a native human speaker or a second analyst will review these.
   - **Response:** Thank you for highlighting this concern. We agree that reliance on automated translation alone may introduce errors or result in the loss of contextual meaning. We have therefore revised the protocol to clarify that automated translation will be used only as an initial aid for screening and data extraction. For all sources meeting the full-text eligibility criteria, the translated content will be reviewed by a team member or collaborator with native or professional proficiency in the source language, or, where appropriate, by a second analyst with sufficient proficiency in that language. This review will verify the accuracy of key extracted data and identify any translation errors, contextual nuances, or meanings that may be missed by automated translation. This clarification has been incorporated into the manuscript (Lines 204-210 and Lines 312-318).
3. Technological Shifts: Educational technology has altered tremendously over the last 16 years - moving from basic low-tech tools to high AI-driven models. The scholars should illustrate how they design to differentiate these kinds of technology in their study.
   - **Response:** Thank you for this valuable observation. We agree that educational technology has evolved substantially over the review timeframe, from low-tech and basic mobile tools to more advanced digital, adaptive, and AI-enabled models, and that this evolution should be reflected analytically rather than treating interventions as technologically homogeneous. Our search strategy includes studies published from 2010 onwards, a timeframe selected to capture the expansion of mobile and low-tech educational interventions in LMICs and that spans substantial changes in educational technology. To ensure that these technological shifts are captured in the review, we have expanded the “Platform/Technology” field in our data-charting form to classify interventions into broad technological categories: (1) low-tech/offline (SMS, radio, and television); (2) basic digital/online (static e-learning platforms, learning management systems, and recorded video content); and (3) advanced/adaptive/AI-enabled (adaptive learning software, AI-enabled tutoring systems, and algorithm-personalized content). The publication year of each included study will also be reported alongside its technological category, allowing the review to descriptively situate intervention models within the broader trajectory of technological change over the review period. This clarification has been incorporated into the Data synthesis plan section of the manuscript (Lines 497-514).
